# Determinants of academic performance in children with sickle cell anaemia

**DOI:** 10.1186/1471-2431-13-189

**Published:** 2013-11-19

**Authors:** Osita U Ezenwosu, Ifeoma J Emodi, Anthony N Ikefuna, Barth F Chukwu, Chidiebere D Osuorah

**Affiliations:** 1 Department of Paediatrics, University of Nigeria Teaching Hospital, Enugu, Nigeria; 2 Child Survival Unit, Medical Research Council UK, The Gambia Unit, Serrekunda, Gambia

**Keywords:** Sickle, Determinants, Academic, Children

## Abstract

**Background:**

Some factors are known to influence the academic performance of children with Sickle Cell Anaemia (SCA). Information on their effects in these children is limited in Nigeria. The factors which influence academic performance of children with SCA in Enugu, Nigeria are determined in this study.

**Methods:**

Consecutive children with SCA aged 5–11 years were recruited at the weekly sickle cell clinic of the University of Nigeria Teaching Hospital (UNTH) Enugu, Nigeria. Their age- and sex- matched normal classmates were recruited as controls. The total number of days of school absence for 2009/2010 academic session was obtained for each pair of pupils from the class attendance register. Academic performance was assessed using the average of the overall scores in the three term examinations of same session. Intelligence ability was determined with Draw-A-Person Quotient (DAPQ) using the Draw-A-Person Test while socio-economic status was determined using the occupational status and educational attainment of each parent.

**Results:**

Academic performance of children with SCA showed statistically significant association with their socio-economic status (*χ*2 = 9.626, p = 0.047), and significant correlation with DAPQ (r = 0.394, p = 0.000) and age (r = -0.412, p = 0.000). However, no significant relationship existed between academic performance and school absence in children with SCA (r = -0.080, p = 0.453).

**Conclusions:**

Academic performance of children with SCA is influenced by their intelligence ability, age and socio-economic status but not negatively affected by their increased school absenteeism.

## Background

Sickle Cell Anaemia (SCA) is the commonest inherited disorder of haemoglobin resulting from the inheritance of mutant haemoglobin genes from both parents [1,2]. While academics is crucial in the development of every human including children [3], some factors may have potential influence on the academic performance of children with SCA. These factors may include:

### School absenteeism

Frequent school absence has been noted in children with SCA [4,5]. It also has been reported as an important predictor of academic attainment [6] as children who are frequently or consistently absent from school tend to perform poorly [7]. This is because multiple, brief or prolonged absences can interfere with the processes of knowledge acquisition as well as other activities. Roby [8] agreed with this and was able to document a statistically significant relationship between students’ attendance and school achievement. This finding was supported by Day and Chismark [9] in the USA who noted poor school performance in children with SCA following frequent school absences due to sickle cell complications.

However, despite the significantly high absence rates reported in SCA children by Ogunfowora *et al.*[4] no significant correlation was found between school absence and academic under-achievement. They argued that SCA may have a more direct impact on the intellectual abilities of some of the affected children through some undetermined mechanism.

### Socio-economic status

Poor school performance has been documented to be high among children from poor socio-economic background [3]. This has been attributed to poor motivation, unsatisfactory home environment and neglect. Other factors contributory to poor school performance include poor housing and nutritional inadequacies. Low socio-economic status was found by Ong and colleagues [10] to contribute to poor academic achievement during early school years. However, available few reports did not document any relationship between parental social status or education and academic performance of children with SCA [4,6,11].

### Intelligence ability

It is known that intelligence (measured as the intelligence quotient or IQ) is one of the important prognostic variables in the academic performance of a child [3]. Children with borderline intelligence (IQ 68–83) or mental subnormality, irrespective of the aetiology, are known to present with poor school performance [12]. Using Wechsler’s Intelligence Scale for Children, WISC, Knight *et al.*[11] found a mean IQ value in SCA children which was 5.6 points lower than their AA controls. Steen and colleagues [13] also assessed the IQ of children with SCA using same tool and noted a significantly below mean value of normative data in full scale, verbal and performance IQ. Similar findings of lower verbal, performance and full scale IQ in children with SCA were also documented by Noll and co-workers [14] as well as Wang and colleagues [15]. Kral and Brown [16] also reported a decreased cognitive function in children with SCA especially those with abnormal transcranial Doppler flow rates.

Children with SCA, therefore, are at risk of poor school performance, since IQ is known to affect school performance [12].

### Age

Certain age groups are more at risk considering SCA morbidity and academic performance. Hawasawi and co-workers [17] demonstrated this in Saudi Arabia when they found the commonest age group affected to be 5-10 year olds while the prevalent cause of admission was Vaso-Occlusive Crisis (VOC). This age group period has also been identified as the critical period for susceptibility to brain infarctions [18] which are increasingly recognized as a major cause of school problems, lower IQ and other neurocognitive deficits [19]. This was in agreement with the study by Pegelow *et al.*[20] who observed that most SCA patients with silent cerebral infarcts had evidence of cerebral damage from the age of 8 years onwards. Moser and colleagues [21] also demonstrated the presence of cerebral infarcts in children with SCA by 6 years with progression over subsequent years.

A progressive decline in neurocognitive and achievement tests with increasing age was also the experience of Wang *et al.*[15], though found in those with normal neuroimaging findings.

### Measures of severity

factors identified as measures of severity of SCA may be clinical or haematological [22]. The clinical factors include number of hospital admissions, clinic visits and painful crisis [11,22]. Haematological factors, on the other hand, include reduced haemoglobin, blood transfusion and reduced HbF [11,22].

Anaemia of any origin may be associated with reduced oxyhaemoglobin saturation [18]. Intellectual impairment in children with SCA is believed to result from a chronic reduction in oxyhaemoglobin saturation of the blood supply to the brain, which underlies the pathophysiologic mechanism of silent cerebral infarction [23]. Dowling and colleagues [23] observed acute silent cerebral infarction in the clinical setting of acute anaemic events in 57% of their subjects and concluded that acute anaemia requiring blood transfusion may be additional risk factors for silent infarcts. An earlier report by Kwiatkowski et *al.*[24] also identified anaemia as a possible risk factor for silent cerebral infarcts in SCA.

Other identified risk factors for silent infarcts include a history of frequent painful events and leukocytosis [25]. Since silent infarcts are recognized as major causes of school problems, low IQ and neurocognitive deficit [19], these measures of severity might possibly have effects on neurocognition and academic performance. This was supported by Steen and colleagues [13] who identified low haematocrit as a significant predictor of cognitive impairment in children with sickle cell disease. Vichinsky *et al.*[26] recently corroborated this finding in adults with SCA.

Limited information is available on the factors associated with the academic performance of children with SCA in Nigeria. This study was therefore carried out to determine the factors that can influence the academic performance of children with SCA in Enugu, Nigeria. It is hoped that the findings from this study will help in formulating policies that will be used in the follow-up clinics of these children. Besides, applying the findings in developing academic programmes for them will improve their academic performance.

## Methods

Primary school-aged children with SCA attending the weekly sickle cell clinic of the University of Nigeria Teaching Hospital (UNTH), Enugu were the study population. Consecutive children with SCA aged 5–11 years who had been in the same primary school for over one academic session during the study period (May – July 2010) were recruited. Necessary data (including age, sex, school, class, name of teacher, medical history, occupation and education of both parents) were obtained from the accompanying parent/caregiver. As part of the medical history, history of past hospital admission(s) and the duration, diagnosis, as well as history of blood transfusion(s) and its frequency during the academic year were documented. The control group were normal classmates of the SCA children as proposed by Richard and Burlew [27]. These controls were next to the subjects in the class register, of same sex and age as the subjects and from similar socio-economic background. The minimum sample size was estimated at 86, based on the estimated prevalence of 50% when prevalence is not known [28]. Ninety children with SCA who satisfied the inclusion criteria were recruited after informed consent were obtained from their parents/caregivers and equal number of pupils were also selected as control group. The home of each of the selected control was visited for informed consent and for the completion of necessary data.

There is no validated academic achievement measure in Nigeria, hence, this study employed the use of school examination report. At the schools, the average score in percentage for each child in each of the three term examinations for 2009/2010 academic session was documented. Average of the three results was calculated as the overall score for the child. This represented the academic performance and was further graded as high (≥ 75%), average (50 – 74%) and low (< 50%). Those with low overall scores were considered as having poor academic performance. This measure has been used previously for the assessment of academic performance of school children [4,29,30]. However, varying standards between individual teachers may affect this measurement strategy.

The total number of days of school absence for 2009/2010 academic session was obtained for each pair of pupils from the class attendance register. High absence was taken as > 12 school days’ absence in the session while low absence was ≤ 12 school days’ absence as recommended by Weitzman *et al.* and described previously [30].

Socio-economic status was determined using the occupation and educational attainment of both parents or their substitutes proposed by Oyedeji as described by Ikefuna and Emodi [31]. Class 1 represented the highest social class and class V the lowest. Each parent was scored separately by finding the average score of the two factors (occupation and educational attainment). The mean of the scores for the father and mother approximated to the nearest whole number was chosen as the social class of the child. The social class was classified into upper (I & II), middle (III) and lower (IV & V) social groups.

In assessing their intelligence ability, the Draw-A-Person Quotient (DAPQ) was determined using the Draw-A-Person Test (DAPT) proposed by Ziler and validated in Nigeria by Ebigbo and Izuora [32]. DAPQ scores less than 75% or 1SD below the average for sex and age group were classified as mental backwardness or dullness while less than 50% or 2SD below age and sex average were classified as mental deficiency [32]. Scores ≥ 75% were classified as normal [32]. DAPT is a measure of visual-spatial-motor conception and execution which has a correlation of 0.62 with Standford-Binet test of intelligence as well as the WISC [32].

Health Research Ethics Committee of UNTH, Enugu approved the study and the Enugu State Ministry of Education gave clearance before the study was commenced. Means were compared using Student’s *t* test while frequencies were compared with Chi squared test. The relationship between two numerical variables was tested using the Pearson’s Correlation Coefficient whereas Chi square was used to test for association between differences in proportions. The level of significance was taken as *p* < 0.05.

## Results

Ninety children with SCA and ninety controls were drawn from 53 primary schools in Enugu. Table 1 shows the age and sex distribution of the subjects and controls. There were 55 (61.1%) males and 35 (38.9%) females (male: female ratio 1.6:1) in each group. The age range was between 5 and 11 years. The mean and standard deviation was 8.88 ± 2.06.

**Table 1:** Age and sex distribution of subjects and controls

**Age**	**Subjects**		**Controls**	
**(Years)**	**M**	**F**	**M**	**F**
5	4	2	4	2
6	10	2	1	2
7	3	3	3	3
8	8	7	8	7
9	3	2	3	2
10	7	9	7	9
11	20	10	20	10
Total	55	35	55	35

Most of the children in this study, 46.7% of the subjects and 48.9% of the controls, belonged to the low socio-economic class. This difference was, however, not statistically significant (*χ*^2^ = 1.46, *p* = 0.834) (Table 2).

**Table 2:** Socio-economic status of the study population

**Socio-economic class**	**Subjects**		**Controls**	
	**No.**	**(%)**	**No.**	**(%)**
Upper	27	(30.0)	21	(23.3)
Middle	21	(23.3)	25	(27.8)
Lower	42	(46.7)	44	(48.9)
Total	90	(100)	90	(100)

*χ*^2^ = 1.14, df = 2, *p* = 0.564.

The mean (SD) of the overall academic scores was 62.71 ± 19.43% for the subjects and 67.47 ± 16.42% for the controls. The difference was not statistically significant (t = -1.776, *p* = 0.077). The frequency distribution of overall academic score ratings of subjects and controls is shown in Table 3. Twenty nine (32.2%) subjects and 15 (16.7%) controls had low performance, and the difference was statistically significant (*χ*^2^ = 5.90, *p* = 0.024). However, 35 (38.9%) and 26 (28.9%) subjects, and 43 (47.8%) and 32 (35.6%) controls were average and high performers respectively. The differences were not statistically significant (*χ*^2^ = 1.45, *p* = 0.229; *χ*^2^ = 0.92, *p* = 0.339 respectively).

**Table 3:** Overall academic score ratings of subjects and controls

	**Subjects**		**Controls**			
**Score rating**	**No.**	**(%)**	**No.**	**(%)**	***χ***^**2**^	***p***
Low performance	29	(32.2)	15	(16.7)	5.90	0.015
Average performance	35	(38.9)	43	(47.8)	1.45	0.229
High performance	26	(28.9)	32	(35.5)	0.92	0.339
Total	90	(100)	90	(100)		

The mean (SD) DAPQ scores for the subjects was 91.41 ± 16.61 while that of the controls was 95.56 ±17.31 and the difference was not statistically significant (t = -1.639, df = 178, *p* = 0.103).

The mean number of days the subjects were absent, 15.85 ± 12.30 days, was significantly higher than the 7.71 ± 8.22 days for the controls (*p* < 0.001) (Table 4). More also shown in Table 4, male and female subjects had higher days of absence than the controls of the same sex and the differences were statistically significant (*p* < 0.001 and *p* = 0.006, respectively). The mean number of days of absence was not significantly different between male and female subjects (t = 0.819, *p* = 0.415) or between male and female controls (t = 0.396, *p* = 0.693).

**Table 4:** Comparison of mean (±SD) number of days of school absence of subjects and controls according to sex

**Days absent from school**							
	**Subjects**		**Controls**				
	**No.**	**Mean (SD)**	**No.**	**Mean (SD)**	**t**	**df**	***p***
Males	55	15.00 (9.10)*	55	7.44 (7.41)**	4.782	108	0.000
Females	35	17.18 (16.18)*	35	8.14 (9.46)**	2.853	68	0.006
All pupils	90	15.85 (12.30)	90	7.71 (8.22)**	5.217	155	0.000

*t = 0.819, *p* = 0.415; **t = 0.396, *p* = 0.693.

**Figure 1:**
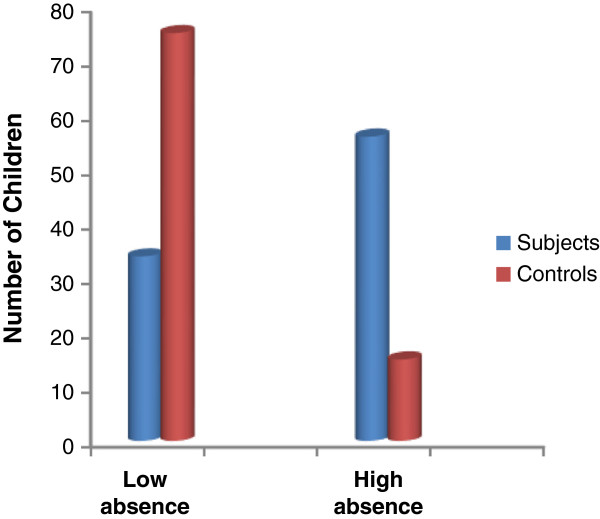
Distribution of subjects and controls by degree of school absence.

The distribution of subjects and controls by degree of school absence is shown in Figure 1. The proportion of subjects in the high absence category 56 (62.2%) was higher when compared to the controls 15 (16.7%). The difference was statistically significant (*χ*^2^ = 39.10, *p* < 0.001). Table 5 shows that 55 (61.1%) subjects were hospitalized during the academic year with an average stay in hospital of 7.8 days per patient. Vaso-occlusive crisis was the commonest reason for hospitalization.

**Table 5:** Predominant diagnoses in the subjects leading to loss of school time

	**No.**	**%**
	**(n = 90)**	
Total number hospitalized	55	61.1
Total no. of hospital admission days	427	
Average duration of hospital stay (days)	7.8	
**Predominant diagnosis on admission**		
Vaso-occlusive crisis	28	51
Malaria	16	29.1
Haemolytic crisis	4	7.3
Sepsis	2	3.6
Septic arthritis	2	3.6
Acute chest syndrome	1	1.8
Haemorrhage	1	1.8
Pneumonia	1	1.8
Total	55	100

The mean academic score of subjects with high degree of school absence (60.57 ± 18.13) was lower than that in those with low degree of school absence (66.26 ± 21.19). However, the difference was not statistically significant (t = 1.356, *p* = 0.179).

The effects of some variables on academic performance of subjects are shown in Table 6. About two-thirds of the low academic scorers (18/29) were high absenters while over half of high academic scorers (14/26) were low absenters. However, there was no statistically significant association between overall score rating and the degree of absenteeism in the subjects (*χ*^2^ = 5.02, *p* = 0.081). Also the correlation coefficient of the relationship between overall academic scores and number of days of absence was negligible and not statistically significant (r = -0.080, *p* = 0.453).

**Table 6:** Effects of variables on the academic performance of children with SCA

**Variables**	**Academic score ratings**			***χ***^**2 **^**( *p *)**	**r (p)**
	**Low**	**Average**	**High**		
**School absence**					
Low	11	9	14	5.023 (0.081)	-0.080 (0.453)
High	18	26	12		
**Socio-economic class**					
Lower	18	13	11	9.626 **(0.047)**	0.313 **(0.003)**
Middle	7	11	3		
Upper	4	11	12		
**DAPQ**					
Low	8	6	3	2.418 (0.298)	0.394 **(0.000)**
Normal	21	29	23		
**Age**					
5–7 yrs	4	7	13	10.946 **(0.027)**	-0.412 **(0.000)**
8–10 yrs	14	14	8		
> 10 yrs	11	14	5		

Bold figures of p-value are statistically significant.

The relationship between academic score ratings and socio-economic class of children with SCA is shown. There was a statistically significant association between academic score ratings and socio-economic class (*χ*^2^ = 9.626, *p* = 0.047). Moreover, there was a statistically significant correlation between overall academic scores and socio-economic class of the subjects (r = 0.313, *p* = 0.003).

As also shown in Table 6, the overall academic scores and DAPQ scores of the subjects had a statistically significant positive linear relationship (r = 0.394, *p* = 0.000).

Academic score ratings of the children with SCA had a statistically significant association with their age. Also, the correlation coefficient, r, between their overall academic scores and age was moderately negative and statistically significant (r = -0.412, *p* = 0.000).

Academic scores in relation to measures of severity of SCA such as transfusion, hospitalization and VOC is shown in Table 7. The subjects who were not transfused had mean (SD) score of 61.95 (18.47%). Subjects with 1 transfusion had higher mean score than those without transfusion while those with 3 transfusions had the highest mean score. The correlation coefficient of the relationship between overall academic scores and number of transfusions was negligible and not statistically significant (r = -0.068, *p* = 0.521).

**Table 7:** Effects of measures of severity on the academic performance of children with SCA

**Severity variables**	**N = 90**	**Academic score**	**t-statistics**	**r statistics**^**†**^
	**n (%)**	**Mean ± SD**	***p-value***	***p-value***
**Blood transfusion**				
0	52 (57.8)	61.95 ±18.47		-0.068
1	25 (27.8)	65.99 ±20.88		*0.521*
2	8 (8.9)	63.50 ±21.15		
3	3 (3.3)	66.07 ±16.06		
4	2 (2.2)	33.20 ± 0.85		
**Hospital admission**				
No	35 (38.9)	61.17 ±16.92	0.598	-0.003
Yes	55 (61.1)	63.69 ±20.96	*0.551*	*0.976*
**Episodes of VOC**				
No	62 (68.9)	62.20 ±18.22	-0.366	
Yes	28 (31.1)	63.83 ±22.18	*0.326*	

^†^Correlation between academic score and measure of SCA severity.

As also shown in Table 7, subjects with past history of hospital admission had a higher mean academic score than those without. However, the difference was not statistically significant (t = 0.598, *p* = 0.551). The correlation coefficient of the relationship between the overall academic scores and duration of hospital admission was negligible and not statistically significant (r = -0.003, *p* = 0.976).

The mean (SD) academic score of the subjects with history of VOC was 63.83 (22.18) while those without VOC had 62.20 (18.22). The difference was not statistically significant (t = -0.366, df = 88, *p* = 0.976). There was no statistically significant association between overall academic score ratings and history of VOC (*χ*^2^ = 0.975, df = 2, *p* = 0.614).

## Discussion

Children with SCA in this study had more frequent school absence than the controls. This finding agrees with previous reports on SCA from the USA [5,6,9] and Nigeria [4]. Reports on other chronic illnesses also noted similar finding [7,33]. The high absence rate in children with chronic ill-health including SCA may be due to many factors. These include frequent routine follow-up visits [34], psycho-emotional disturbances [35], and recurrent crises resulting in frequent hospitalization [34]. Pain was the most common symptom contributing to absenteeism [9,34]. This is supported by the results of this study which showed that VOC constituted the commonest reason for hospitalization of children with SCA. SCA children with pain not requiring hospital admission can also experience school absence [36]. The extent of this was not explored in this study. Apart from VOC, in this study, another contributor to school absenteeism in children with SCA is malaria. This was not a documented contributory factor in school absenteeism in the other published studies outside Nigeria [5,9,34], possibly because malaria is rare to non-existent in these areas while it is endemic in Nigeria [37].

Though Schatz [6] argued that school absence in SCA patients is an important predictor of academic attainment, while Moonie *et al.*[7] believed that children who are frequently absent from school tend to perform poorly, no association was found between academic performance and school absence in this study. Ibekwe *et al.*[33] also noted a similar lack of association between academic performance and absenteeism in children with epilepsy. It is possible that these children found it necessary to make up for lost time, thereby making up for academic lapses that might be related to their absence from school.

This study found no significant difference between the mean overall academic score of subjects and controls. This corroborates the findings of Ogunfowora and colleagues [4] in Nigeria. In spite of the comparable overall academic score of SCA patients and controls, a higher proportion of low performance children was found among the SCA patients. This is consistent with previous findings [4,6]. The under-achievement in children with SCA may be unrelated to higher rate of school absence as there was no significant difference between the mean scores of high absenters and low absenters. More so, no association was found between academic performance and school absence in SCA patients and the relationship between the two variables was negligible.

There was a significant positive linear relationship between academic performance and DAPQ scores of SCA patients. This is similar to the experience of other workers [3,12]. Thus, as has been suggested [9], intelligence ability scores may be suitable guide in the proper placement of school children at the beginning of their education.

Measures of severity of SCA such as blood transfusions, history and duration of admission, and VOC, individually had no effect on the academic performance of the subjects. This is in agreement with the findings of Knight *et al.*[11] in Jamaica. In contrast, however, Steen *et al.*[13] and Vichinsky *et al.*[26] in the USA found a relationship between a haematological index (low haematocrit) and cognitive impairment in children and adults with SCA respectively. The contrast between this and our findings may be due to the fact that the haematological index we studied was blood transfusion, which may improve cerebral blood flow, oxygenation, and neurocognitive function in children with SCA [38]. Researchers on other chronic diseases have reported that anaemia is predictive of poor neurocognitive performance while increasing haemoglobin levels improved the performance [39,40].

The decline of academic performance with increasing age in children with SCA is consistent with the finding of Wang *et al.*[15]. This could be attributed to greater level of network of activated brain regions during processing tasks and mental activities exhibited by younger children than the older ones [41]. Another plausible explanation could be that the older children are faced with more problems including burdensome homework, over-scheduled activities, and television viewing etc., which might cause sleep disturbances with consequent lower cognitive function [42,43].

The results demonstrated a relationship between academic performance and socio-economic class in children with SCA. This trend has been documented earlier [6] and it is in keeping with previous observations that academic under-achievement was generally more common among children of poorly educated parents in the lower socio-economic classes [10,44]. Unlike other parents from low socio-economic classes, parents of SCA patients from low socio-economic classes may be unable to provide extra academic facilities to boost their performance because of the depletion of the family’s resources in caring for a chronically ill child.

## Conclusions

Academic performance of primary school children with SCA is not affected by their school absenteeism. However, it declines with increasing age and has an association with intelligence ability and socio-economic status. Since the academic performance of SCA patients reduces with increasing age, extra academic programme is required for these children as they advance in age. Also regular evaluation of their intelligence ability in the follow-up clinics is important so as to detect any early deviation from normal. Such deviations may require remedial measures/interventions.

## Competing interests

The authors declare that they have no competing interests.

## Authors’ contributions

OUE carried out the design of the study, acquisition of data, analysis and interpretation of data and drafted the manuscript. IJE conceived of the study, participated in the design and helped in its draft. ANI participated in the design of the study and its analysis and interpretation. BFC helped in drafting of the manuscript. CDO participated in analysis of data and review of the manuscript. All authors read and approved the final manuscript.

## Pre-publication history

The pre-publication history for this paper can be accessed here:

http://www.biomedcentral.com/1471-2431/13/189/prepub
